# Maternal anthropometry and feeding behavior toward preschool children: association with childhood body mass index in an observational study of Chilean families

**DOI:** 10.1186/1479-5868-6-93

**Published:** 2009-12-29

**Authors:** José Luis Santos, Juliana Kain, Patricia Dominguez-Vásquez, Lydia Lera, Marcos Galván, Camila Corvalán, Ricardo Uauy

**Affiliations:** 1 Departamento de Nutrición, Diabetes y Metabolismo. Facultad de Medicina, Pontificia Universidad Católica de Chile, Santiago, Chile; 2 Instituto de Nutrición y Tecnología de los Alimentos (INTA), Universidad de Chile, Santiago, Chile; 3 Departamento de Salud Pública, Facultad de Medicina, Universidad de Chile. Santiago, Chile

## Abstract

**Background:**

A better understanding of the link between eating behavior and maternal feeding practices with childhood and maternal weight status is of great interest.

**Objective:**

To assess the association between childhood anthropometric measures with mothers' Body Mass Index (BMI) and their feeding practices toward preschool children in Chile.

**Methods:**

1029 children (504 boys, 4.3 ± 0.3 years) and their mothers were selected from public nurseries located in low income neighborhoods in Santiago. Mothers' BMI, children's BMI and waist-to-height ratios were registered. Maternal feeding practices towards their children's nutritional habits were measured using an adapted version of the Child Feeding Questionnaire (CFQ).

**Results:**

We found a direct correlation (p < 0.001) between children's BMI z-score and their mothers' BMI, both in boys (Spearman rho = 0.26) and girls (rho = 0.30). A direct association was also found between children's BMI z-score with scores of the subscale "concern for child's weight" (Spearman rho = 0.26 in boys and rho = 0.37 in girls; p < 0.001) and "food restriction" (rho = 0.19 in boys and rho = 0.27 in girls; p < 0.001). A reverse significant association was found between children's BMI z-score with scores of "pressure to eat" (rho = -0.30 in boys and rho = -0.36 in girls; p < 0.001). Analyses of the combined categories of childhood obesity and/or maternal obesity showed an important influence of children's weight status on CFQ scores.

**Conclusion:**

Mothers' BMI and children's BMI z-scores are highly correlated. We found significant associations between mothers' behaviour subscales and children's BMI z-score. It is not possible to establish a causal link between mother's CFQ scores and children's nutritional status, given the cross-sectional nature of this study and the bidirectional influences that exist between mothers and their children.

## Background

During the last decades, Chile has experienced a nutrition transition, with a rapid decline in undernutrition and an increase in the prevalence of obesity as a consequence of social changes leading to a rise in consumption of high-calorie foods and sedentary habits [1,2]. This transition has occurred at a much faster rate than in other countries [3]. As a consequence, it is conceivable that attitudes and practices of many Chilean mothers on feeding and nutrition are somehow still dominated by a sense of protection against childhood undernutrition instead of the prevention of obesity. In this context, it is important to note that obesity is currently the most frequent nutritional disease in Chilean children, with a prevalence that has tripled over the past 15 years; it is currently 21% among 6 year-old children [3,4]. Since obesity prevention should start early in life, it is important to determine the factors that influence weight excess and eating behavior in preschool children [5]. It is generally accepted that parents' practices about feeding, especially mother's practices, are important factors to consider in order to achieve a better nutrition during childhood [6-10]. However, it is still difficult to define the best parental strategy in relation to childhood feeding, especially under the strong social influences that characterize the "obesogenic" environment.

Research about the effect of food intake on childhood obesity has traditionally focused on the amount and type of foods usually consumed. However, a better understanding of the link between eating behavior and maternal feeding practices with childhood obesity is also of interest. Computed standardized questionnaires using behavior scores covering different eating dimensions such as the Child Eating Behavior Questionnaire and the Three-factor Eating Questionnaire can be used for this purpose [10]. The use of psychometric tools to assess how eating practices and attitudes of parents, measured for example through the Child Feeding Questionnaire (CFQ), may influence eating behavior and the nutritional status of children can yield valuable information to better define successful intervention strategies [11].

Individual feeding habits and practices are acquired in early childhood, representing behavior traits that may change over time according to personal experiences throughout the lifetime [10]. Besides the role of the social context, it is also assumed that familial factors, both common environment and genetic inheritance, have an important influence on food intake and eating behavior, which is also linked to childhood obesity [6-9]. Although it is generally accepted that parental feeding styles have a relevant influence on children eating behavior, more evidence is required from studies covering different obesity status in children and/or parents, as well as groups from developing countries. Results from studies on parental feeding styles may provide useful information that can serve to develop guidelines for parents that are consistent with healthy diets and promote children's ability to self-regulate intake [12-14].

There is a scarcity of studies in Latin America on the relation between maternal feeding styles or maternal Body Mass Index (BMI) with children's weight status. Thus, the aim of this study was to assess the association between maternal feeding practices and BMI with anthropometric measures related to obesity in Chilean preschool children.

## Subjects and Methods

### Subjects

During 2006, a sample of 1195 preschool children with ages ranging from 3.1 to 5.8 years (mean = 4.3 years; standard deviation = 0.3 years) was selected from fifty-four nursery schools belonging to the National Association of Day Care Centres (JUNJI, acronym for Junta Nacional de Jardines Infantiles) located in Santiago. JUNJI is a nationwide program offering free education and food to middle-low and low income children for 4 or 8 hours/day. Consequently, the socioeconomic profile of the participants of this study is relatively uniform. The inclusion criteria for the study were: a) children attending JUNJI centres located in 6 representative counties of Santiago, b) singleton births with weight > 2500 g and c) absence of physical or psychological conditions that could compromise growth. The selection of children in this cross sectional survey was not random, since all children fulfilling the inclusion criteria were included. No significant differences were detected in terms of age, gender, birth anthropometry, weight and height among the children who participated compared to those not enrolled, according to JUNJI information.

As expected, the mother was the main caretaker for the vast majority of the children. After discarding data from 31 families in which the main caretaker was not the mother, families with outliers for anthropometric data (see "Anthropometric measurements") or families with incomplete measurements, a total sample of 1,029 child-mother pairs provided complete information on child and mother's BMI. Among these 1,029 child-mother pairs, more than 99% also provided information on questionnaires on maternal feeding practices towards their son/daughter. The mean age and standard deviation of children were 4.3 ± 0.3 years (range: 3.3 - 5.7) (504 boys and 525 girls). The mean age and standard deviation of mothers were 31.8 ± 7.0 years (range: 19 - 50). The study protocol was approved by the Executive Director of the JUNJI Program and the Institute of Nutrition and Food Technology (INTA) Ethics Committee of the University of Chile. Written informed consent was obtained from all parents or guardians.

### Anthropometric measurements

Weight and height of children and their mothers were directly measured by trained personnel using a standardized protocol, in order to calculate Body Mass Index (BMI) as weight (Kg.) divided by height squared (in meters). Additionally, waist circumference was determined in the children in order to calculate their waist-to-height ratios. BMI z scores were calculated using the WHO 2006 Growth Standards as a reference [15]. Cut-off points follow WHO criteria: low-weight (BMI-z < -1), normal weight (BMI z-score from -1 to +1), overweight (BMI z-score from +1 to +2) and obesity (BMI z-score >+2) http://www.who.int. There were no children with BMI-z < -2 and less than 1% of the measurements were excluded from the analyses after being flagged as outliers (BMI Z > and < 5).

### Maternal feeding practices

Mothers were interviewed at their homes by trained personnel. The identification of the type and level of maternal control over child feeding was obtained through the application of the Child Feeding Questionnaire (CFQ) [16]. The psychometric proprieties of CFQ and its construct validity in relation to energy intake and weight status have been previously reported [17,18] and evaluated in different populations [19-21]. Revised version of the original CFQ [10] includes 31 items measuring seven sub-scales of eating behavior. In this study, we have focused on five subscales of the CFQ (Table 1). The CFQ had been adapted previously for the Chilean population by a process of direct and reverse translation, with a subsequent analysis of a pilot study of 10 child-mother duos, in which we assessed the comprehension and cultural appropriateness of the test for the Chilean context. All subscales showed an internal consistency higher than 0.6 as measured with the Cronbach-alpha statistic (Table 1).

**Table 1:** Mother's child feeding practices measured by the Child Feeding Questionnaire for Chilean preschool children

Subscale	Definition^1^	Items	Range	Cronbach-alpha
**Perceived responsibility**	Degree of mother's responsibility for feeding her child, determining portion sizes, and providing a healthy diet.	3	1 (low feelings of responsibility) to 5 (high feelings of responsibility)	0.75

**Concern about child's weight**	Degree of mother's concern over her child becoming over-weight	3	1 (unconcerned) to 5 (highly concerned)	0.72

**Restriction**	Degree of mother's control over child's eating by restricting access to palatable foods (type and amount).	8	1 (low restriction) to 5 (high restriction)	0.65

**Pressure to eat**	Degree of mother's encouragement on her child to eat. For e.g, finishing everything on the plate.	4	1 (low levels of pressure) to 5 (high levels of pressure)	0.61

**Monitoring**	Degree of mother's awareness of child's consumption of sweets, snacks and other high-fat foods.	3	1 (never) to 5 (always)	0.82

Each item was answered in a Likert-type scale with possible scores from one to five (Table 1). The standard scores for each subscale were calculated as average results of individual scores divided by the number of items in that subscale.

### Statistical analysis

Summary statistics for CFQ scores are shown as percentiles 25, 50 and 75. Descriptive statistics for continuous anthropometric variables are shown as means and standard deviations. The null hypothesis of normality was rejected for some of anthropometrical variables. Regarding CFQ scores, only cognitive restriction subscale scores followed normality in a combined test of skewness and kurtosis. Therefore and given the nature of scores based on Likert-type items, we finally decided to use non-parametric statistical tests based on ranks (Mann-Whitney and Kruskall-Wallis tests) to assess the differences across study groups. We also decided to use Spearman non-parametric coefficients for all pair-wise correlations between variables. The crude odds ratio relating childhood obesity and mother's obesity was also computed. Additionally, logistic regression analyses were carried out in both sexes, with children's obesity as dependent variable and maternal feeding practices (coded as 1 for score > 4 and 0 for scores ≤ 4), and maternal obesity as independent variables.

The rationale for the initial sample size calculations was based on detecting significant correlation coefficients between the mother's BMI and BMI z-scores of the children. The achieved sample size provided sufficient statistical power (above 90%; alpha = 0.05) to detect significant correlations between anthropometric variables in child-mother duos. Statistical analyses were carried out with the STATA 10.0 package http://www.stata.com.

## Results

In the children, the prevalence of low-weight was 1.7% (95% confidence interval 1.0 - 2.6%); normal weight: 54.0% (95% CI 50.9 - 57.1%); overweight: 29.7% (95% CI 27.0 - 32.6%), and obesity: 14.6% (95% CI 12.5 - 16.9%). The nutritional status of mothers was as follows: underweight (BMI < 18.5 Kg./m^2^), 0.78% (95% CI 0.3 - 1.5%); normal weight (18.5-24.9 Kg./m^2^), 38.9% (95% CI 35.9 - 41.9%); overweight (25.0-29.9 Kg./m^2^), 35.6% (95% CI 32.5 - 38.5%); and obesity (BMI ≥ 30 Kg./m^2^), 24.7% (95% CI 22.3 - 27.6%).

Table 2 shows the general description of the subjects participating in this study. No significant differences in anthropometric variables, age and CFQ scores were found by gender, except for a higher height and BMI in boys, with no significant differences in BMI z-scores. We found a significant direct correlation (p < 0.001) between children BMI z-score with mother's BMI, both in boys (Spearman rho = 0.26) and girls (rho = 0.30). Similar significant correlations (p < 0.001) were found between child waist-to-height ratio and mother's BMI (rho = 0.20 in boys and rho = 0.22 in girls). The cross-sectional odds ratio relating childhood obesity (BMI z-score ≥ 2) and mother's obesity (BMI ≥ 30 Kg./m^2^) in our study was estimated as 2.17 (95% CI: 1.48 - 3.17).

**Table 2:** Main characteristics of the participants in this study

	Boys (n = 504)	Girls (n = 525)	P-value
**Age & Anthropometry**			
**Age (years)**	4.31 (0.34)	4.30 (0.32)	0.44
**Weight (Kg.)**	18.4 (2.7)	18.2 (2.7)	0.17
**Height (cm.)**	104.6 (4.5)	103.9 (5.8)	0.02
**BMI (Kg./m^2^)**	16.5 (1.7)	16.8 (1.7)	0.98
**BMI z score**	1.0 (1.1)	1.0 (0.83)	0.24
**Obesity (% BMI z-score ≥ 2)**	16.5%	12.8%	0.09
**Waist circumference (cm.)**	53.7 (4.0)	53.3 (4.0)	0.04
**Waist-to-Height ratio**	0.51 (0.03)	0.51 (0.04)	0.50
**Maternal age (years)**	31.4 (6.8)	32.1 (7.1)	0.20
**Maternal BMI (Kg./m^2^)**	27.3 (5.3)	26.9 (4.9)	0.32
**CFQ Scores**			
**Perceived responsibility**	4.7 (4.3-5.0)	5.0 (4.3-5.0)	0.99
**Concern over child's weight**	4.3 (3.7-4.7)	4.3 (3.7-5.0)	0.13
**Restriction**	2.8 (2.4-3.1)	2.8 (2.3-3.1)	0.30
**Pressure to eat**	3.8 (3.0-4.3)	3.8 (3.0-4.3)	0.33
**Monitoring**	4.3 (3.7-5.0)	4.3 (3.7-5.0)	0.98

Anthropometric and socio-demographic variables are displayed as mean (standard deviation). CFQ scores are shown as median (percentile 25 - percentile 75). Differences by gender were assessed through the Mann-Whitney test

Table 3 shows Spearman correlation coefficients for the association between anthropometric variables of the child-mother duo and CFQ scores reported by the mother. We found a significant direct association (p < 0.0001) between children BMI z-score with the subscale "Concern about Child's weight" (rho = 0.26 in boys and rho = 0.37 in girls) and "Restriction" (rho = 0.19 in boys and rho = 0.27 in girls), as well as a reverse significant associations with "Pressure to eat" (rho = -0.30 in boys and rho = -0.36 in girls). Similar association, although of lower magnitude, were detected for the association between such CFQ subscales and children's waist-to-height ratio. In all, the significant association using CFQ scores, the magnitude of the Spearman correlation coefficient was higher in girls than in boys. No significant differences were found in the relation between BMI z-scores of the children and the CFQ subscale of "Monitoring". Although some associations between BMI z-scores and the subscale "Responsibility perceived" achieved statistical significance, this relation was dominated by low-magnitude correlations (rho absolute values lower or around 0.1). The correlations of CFQ scores with mother's BMI were in general non-significant or slightly significant, except for the significant direct association between mother's BMI and the subscale "Concern about child's weight" (the higher the mother's BMI, the higher the degree of child's weight concern) and the reverse correlation for "Monitoring" (the lower the mother's BMI, the higher the degree of monitoring). The logistic regression analysis with the presence of obesity in mothers as the dependent variable and "concern for child weight", gender and children's weight as independent variables, showed that the crude significant relation between Mother's BMI and concern for child weight was no longer significant after adjusting for child weight status (p = 0.35).

**Table 3:** Correlation analysis between CFQ scores and anthropometric variables of preschool children and their mothers

	Mothers' BMI		Children BMI z-score		Children waist-to-height ratio	
	
CFQ scores	rho	P-value	rho	P-value	rho	P-value
**Perceived responsibility**						
**Boys**	0.10	0.02	-0.03	0.43	-0.02	0.70
**Girls**	0.05	0.25	-0.08	0.07	-0.12	0.005
						
**Concern over child's weight**						
**Boys**	0.11	0.02	0.26	< 0.001	0.23	< 0.001
**Girls**	0.17	< 0.001	0.37	< 0.001	0.24	< 0.001
						
**Restriction**						
**Boys**	0.07	0.13	0.19	< 0.001	0.15	< 0.001
**Girls**	0.10	0.02	0.27	< 0.001	0.23	< 0.001
						
**Pressure to eat**						
**Boys**	-0.05	0.28	-0.30	< 0.001	-0.19	< 0.001
**Girls**	-0.10	0.03	-0.36	< 0.001	-0.27	< 0.001
						
**Monitoring**						
**Boys**	0.05	0.30	0.08	0.06	0.08	0.08
**Girls**	0.04	0.33	0.02	0.64	-0.01	0.88

Spearman correlation coefficient (rho)

Table 4 shows the results of the multivariate logistic regression analysis in both sexes, with children's obesity as the dependent variable and maternal feeding practices (coded as 1 for score > 4 and 0 for scores ≤ 4) and maternal obesity as independent variables. Results of table 4 essentially provide the same information as the non-parametric crude analysis based on Spearman correlations.

**Table 4:** Multivariate odds ratios for the association between children's obesity and CFQ binary categories

	Odds Ratio	95% CI	p-value
**Girls**			
**Perceived responsibility**	0.5	0.3-0.9	0.02
**Concern over child's weight**	10.9	4.6-25.8	<0.001
**Restriction**	5.0	0.8-31.1	0.09
**Pressure to eat**	0.2	0.06-0.4	<0.001
**Monitoring**	0.8	0.5-1.3	0.39
			
**Boys**			
**Perceived responsibility**	0.9	0.4-1.7	0.71
**Concern over child's weight**	4.1	2.4-7.2	<0.001
**Restriction**	15.1	2.9-77.7	0.001
**Pressure to eat**	0.4	0.2-0.8	0.005
**Monitoring**	1.7	1.0-2.7	0.05

Logistic regression analysis were carried out in both sexes, with children's obesity as dependent variable and each maternal feeding practice (coded as 1 for score > 4 and 0 for scores ≤ 4) as independent variable, adjusting by maternal obesity.

**Figure 1:**
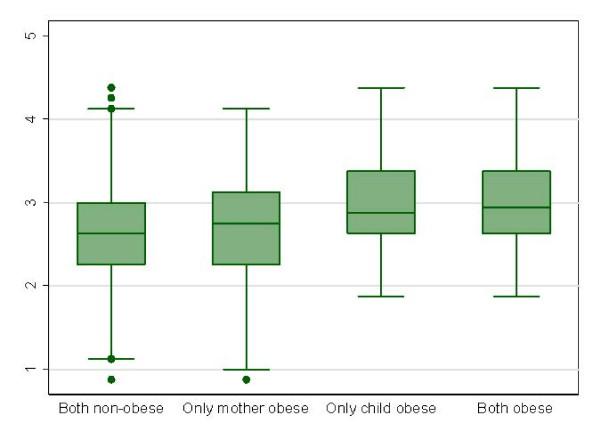
**Box-plot of "Restriction" scores of the Child Feeding Questionnaire according to the combined nutritional status in preschool Chilean children and their mothers**. Kruskal-Wallis test P < 0.001.

**Figure 2:**
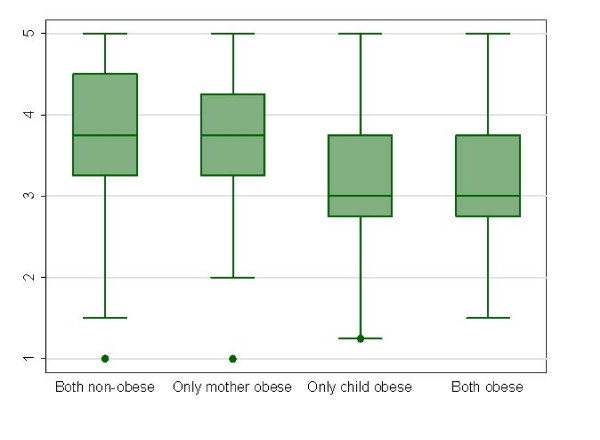
**Box-plot of "Pressure to eat" scores of the Child Feeding Questionnaire according to the combined nutritional status in preschool Chilean children and their mothers**. Kruskal-Wallis test P < 0.001.

On the other hand, the effect of the combined presence of obesity in the child (BMI z-score ≥ 2) and the mother (BMI ≥ 30 Kg./m^2^) on CFQ scores showed significant differences among study groups (both non-obese, only obese child, only obese mother, both obese) for scores of "Perceived Responsibility" (p = 0.004), "Concern about child's weight" (p < 0.001), "Restriction" (p < 0.001), "Pressure to eat" (p < 0.001), without finding significant differences for scores in "Monitoring" (p = 0.11). As shown in Figures 1 and 2, obesity in the child is the main determinant of the significant differences found for "Restriction" scores (Figure 1) and "Pressure to eat" scores (Figure 2), as well as in the differences for "Perceived Responsibility" and "Concern about Child's weight" (data not shown).

## Discussion

Children in this study were, on average, overweight; this coincides with results obtained from a study on the nutritional situation of Chilean preschool children from Santiago [4]. This study confirms the existence of the well-known cross-sectional correlations between maternal BMI and childhood BMI z scores, reflecting that the family influence, characterized by both shared genetic background and environment, has an important impact on obesity traits [22]. Supporting this idea, Reilly et al. [23] found a very strong association between parental and childhood obesity in the Avon Longitudinal Study of Parents and Children (ALSPAC) prospective study, with odds ratios above 10 for childhood obesity in those with both obese parents, or above 4 if only the mother was obese. We also have detected higher child-mother BMI correlations in the subset of girls compared to boys, by comparing the magnitude of Spearman correlation rho values for daughter-mother duos compared to son-mother duos. In this respect, it has been reported a greater similarity of anthropometric measures and dietary intake patterns of daughters and mothers compared to those of sons and fathers [24]. Likewise, we have also detected a high correlation between maternal BMI and waist-to-height ratio in children. In this context, it is important to note that the waist-to-height ratio has been reported as an adequate, accurate and relatively non-age-dependent index to assess abdominal obesity status in children [25].

Several significant correlations between CFQ scores and childhood BMI z scores have been observed in this study, in accordance to that observed by other authors [9,11,18,26,27]. In general, the magnitude of the association between CFQ scores and child BMI z scores was higher in the duos girls-mothers than in duos boys-mothers, with the exception of the correlations for the subscale "monitoring". The observation of a higher BMI z-score in the child with a higher degree of "concern for child's weight" in the mother may be due to the awareness of mothers on childhood obesity risks. In this sense, the highest scores for this CFQ subscale have been estimated in obese mothers. Apart from methodological issues that differ from study to study, the observation of such a correlation has been found to be highly variable in different populations, with some authors suggesting a direct association, reverse association or no-association [9,28-30]. It has also been observed that the higher the degree of maternal practices towards child eating restriction, the higher the BMI z-score of the child. This specific association may refer to the attempts of mothers to limit energy intake specifically in overweight or obese children. Additionally, these results are somehow concordant with previous studies suggesting that restriction imposed by parents is associated with higher BMI and a higher fat consumption [31,32]. However, this association has not been observed in other studies [33,34]. On the other hand, the higher levels of "child weight concern" and "Restriction" shown in girls could be explained by social pressure for body image [35]. Conversely, the observation of a correlation of lower BMI z-score s in the child with a higher degree of "pressure to eat", may be related to the mothers' practices against childhood undernutrition [11,30,31]. Multivariate odds ratios with childhood obesity as dependent variable (Table 4) reinforce the conclusions obtained in the non-parametric correlation analysis. On the other hand, the exploration of combined categories of obesity in the child and/or in the mother, indicates that obesity in the child, regardless of mother's obesity, is associated with higher scores of "Restriction" and lower scores in "Pressure to eat" (Figures 1 and 2). We have not detected a significant differential pattern of maternal feeding practices depending on the gender of the child (Table 2).

There are some limitations to the interpretation of correlation coefficients in this study that relate to its cross-sectional nature. In this sense, significant associations detected in this study may in fact be due to reverse causation process in which the disease (childhood obesity) caused mothers to change their behavior in relation to childhood feeding. It is important to point out that the effects observed are probably bidirectional, with parents influencing children's eating and viceversa, being parents behavior also conditioned by children's weight status [36]. On the other hand, there are also concerns regarding social desirability issues, since parents could be inclined to report on how they ought to behave instead of rating their actual behavior. Finally, availability of foods for families of low or middle-low socioeconomic status such as the ones included in this study may also mediate the association between parental feeding practices and intake of the children. Given the complexity of behavioral variables and their relation to intake and nutritional status, it is important to recognize that numerous variables were not controlled for in our study.

In conclusion, we have observed a highly significant correlation between the BMI of mothers and the BMI z-scores of their preschool children in Chilean families. Moreover, there are significant associations between mothers' behaviour subscales and children's BMI z-score (positive for "concern for child's weight" or "restriction" and negative for "pressure to eat"). It is not possible to establish a causal link between mother's CFQ scores and children's nutritional status given the cross-sectional nature of this study and the bidirectional influences that exist between mothers and their children.

## Competing interests

The authors declare that they have no competing interests.

## Authors' contributions

JLS participated in the original formulation of the research project. He was in charge of the statistical analysis, of writing the present manuscript together the rest of co-authors. JK was the leader of this research project, obtaining funding for its implementation, coordinating all aspects of the investigation from study design to data interpretation. She actively revised the manuscript. PDV was in charge of preliminary statistical analysis, especially in relation to the CFQ data. She did the literature search on this topic. LL kept the validity and veracity of the whole dataset. She revised the manuscript. MG was responsible for the field work and revised the manuscript. CC participated in the field work writing and revised this manuscript. RU participated in the formulation of the research project, participated in the field work, evaluated the results and reviewed the manuscript. All authors have read and approved the final version.
